# Hippocampal metabolism of amino acids by L-amino acid oxidase is involved in fear learning and memory

**DOI:** 10.1038/s41598-018-28885-x

**Published:** 2018-07-23

**Authors:** Kento Usuda, Takahiro Kawase, Yuko Shigeno, Susumu Fukuzawa, Kazuki Fujii, Haolin Zhang, Takamitsu Tsukahara, Shozo Tomonaga, Gen Watanabe, Wanzhu Jin, Kentaro Nagaoka

**Affiliations:** 1 United Graduate School of Veterinarian Science, Gifu University, Gifu, Gifu Japan; 2 Laboratory of Veterinary Physiology, Department of Veterinary Medicine, Tokyo University of Agriculture and Technology, Fuchu, Tokyo Japan; 3 Kyoto Institute of Nutrition and Pathology, Tsuzuki, Kyoto Japan; 4 Laboratory of Benno, RIKEN Innovation Center, Wako, Saitama Japan; 5 Life Science Research Center, Toyama University, Toyama, Toyama Japan; 6 College of Biological Science and Technology, Beijing Forestry University, Haidian, Beijing China; 7 Division of Applied Biosciences, Graduate School of Agriculture, Kyoto University, Kyoto, Kyoto Japan; 8 Institute of Zoology, Chinese Academy of Sciences, Chaoyang, Beijing China

## Abstract

Amino acids participate directly and indirectly in many important biochemical functions in the brain. We focused on one amino acid metabolic enzyme, L-amino acid oxidase (LAO), and investigated the importance of LAO in brain function using *LAO1* knockout (KO) mice. Compared to wild-type mice, *LAO1* KO mice exhibited impaired fear learning and memory function in a passive avoidance test. This impairment in *LAO1* KO mice coincided with significantly reduced hippocampal acetylcholine levels compared to wild-type mice, while treatment with donepezil, a reversible acetylcholine esterase inhibitor, inhibited this reduction. Metabolomic analysis revealed that knocking out *LAO1* affected amino acid metabolism (mainly of phenylalanine [Phe]) in the hippocampus. Specifically, Phe levels were elevated in *LAO1* KO mice, while phenylpyruvic acid (metabolite of Phe produced largely by LAO) levels were reduced. Moreover, knocking out *LAO1* decreased hippocampal mRNA levels of *pyruvate kinase*, the enzymatic activity of which is known to be inhibited by Phe. Based on our findings, we propose that *LAO1* KO mice exhibited impaired fear learning and memory owing to low hippocampal acetylcholine levels. Furthermore, we speculate that hippocampal Phe metabolism is an important physiological mechanism related to glycolysis and may underlie cognitive impairments, including those observed in Alzheimer’s disease.

## Introduction

**Figure 1:**
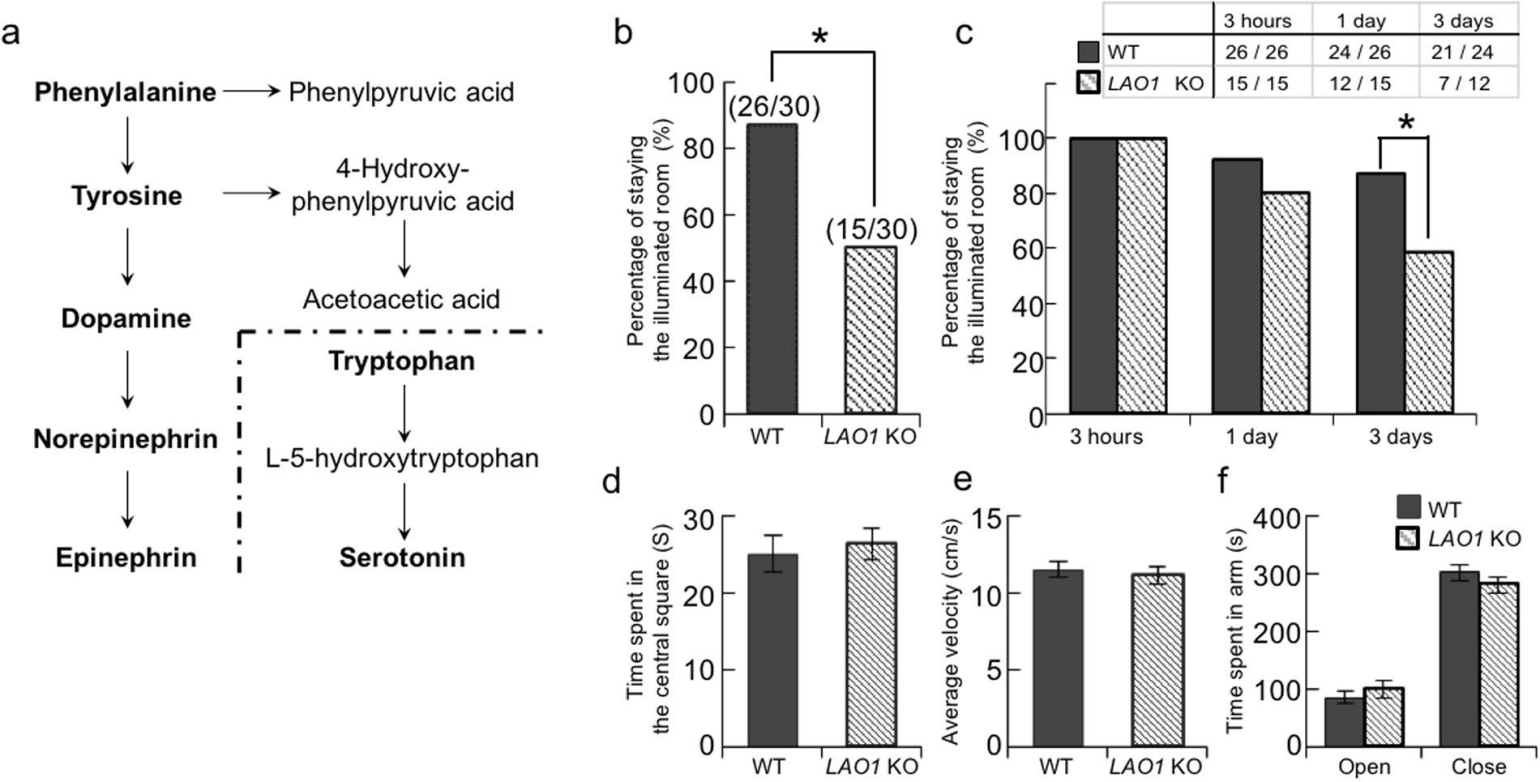
Impairment of fear learning and memory in LAO1 KO mice. (**a**) Schematic diagram showing the biosynthesis of catecholamines from phenylalanine and serotonin from tryptophan. (**b**,**c**) Passive avoidance test. Adult male WT (n = 30) and *LAO1* KO (n = 30) mice received foot shocks in the dark compartment on the first day. The results represent the percentage of time spent avoiding the dark compartment by the mice at 3 h, 1 day and 3 days after the foot shock. These mice were also compared in two tests for emotional- and anxiety-like behaviour, namely the open field test (male WT mice: n = 22, male *LAO1* KO mice: n = 22) (**d**) time in centre and (**e**) average velocity) and elevated plus maze (male WT mice: n = 22, male LAO1 KO mice: n = 22) (**f**).

Amino acids play exclusive roles in the synthesis of a large number of biologically important compounds, such as proteins, peptides, lipids, and vitamins. In addition, free amino acids and their metabolites are involved in many physiological functions, especially synaptic transmission, where they serve as neurotransmitters and neuromodulators. The aromatic amino acids tryptophan (Trp), tyrosine (Tyr), and phenylalanine (Phe) are the biosynthetic precursors for the neurotransmitters serotonin, dopamine, and norepinephrine (Fig. 1a)^1,2^. Moreover, it has been shown that the expression levels of other amino acids, such as taurine, aspartic acid, and glutamic acid, are higher in the brain than they are in other tissues and that these amino acids contribute to learning and memory processes^3^.

The hippocampus is a crucial structure in the brain for learning and memory^4,5^. For example, contextual fear memory is hippocampus-dependent and is regulated by glutamic acid, calcium calmodulin-dependent kinase 2, brain-derived neurotrophic factor, and other molecules related to synaptic plasticity^6,7^. Hippocampal cholinergic nerve terminals are also important for memory function. Specifically, the release of acetylcholine enhances memory by modulating the induction of synaptic plasticity^8,9^. Additionally, reductions in acetylcholine levels are strongly related to neurodegenerative diseases, such as Alzheimer’s disease (AD)^10^. The drug donepezil, which is a reversible acetylcholine esterase (AChE) inhibitor, has been proven to be effective in improving memory deficits in both animal models of and patients with AD^11–14^. Recently, it was suggested that the amino acid metabolic cascade is involved in the regulation of the acetylcholine level in the hippocampus^15,16^; however, the specifics of this relationship remain unclear.

The metabolism of amino acids is highly controlled by biochemical and physiological mechanisms that lead to relatively stable levels of free amino acids in the blood and tissues^17,18^. Excess amino acids are catabolised in the reactions of gluconeogenesis and are used to synthesise acetyl coenzyme A and several metabolites involved in the tricarboxylic acid cycle^19,20^. Various diseases caused by abnormal amino acid metabolism are known to affect brain function. For example, maple syrup urine disease, which is due to an inborn error in metabolism caused by decreased activity of the branched-chain keto acid dehydrogenase complex, is characterised by psychomotor delays, and mental retardation^21,22^. Phenylketonuria (PKU), one of the most common metabolism-related diseases in humans, is caused by a recessively inherited phenylalanine hydroxylase (Pah) deficiency and leads to profound cognitive disability owing to Phe accumulation^23,24^. Several recent studies also provided evidence for a possible link between the accumulation of Phe and AD. One study showed that the serum concentration of Phe was elevated in patients with AD when compared to healthy individuals^25^ while another noted the assembly of Phe into toxic fibrils with an amyloid-like morphology in brain tissues from patients with PKU^26^. However, the exact mechanisms underlying the brain dysfunction that is associated with abnormal amino acid metabolism remain poorly understood.

L-amino acid oxidase (LAO, EC 1.4.3.2) is an amino acid metabolic enzyme that catalyses the oxidative deamination of specific L-amino acids, such as Phe, Tyr, and leucine, and converts them into keto acids, ammonia, and hydrogen peroxide (H_2_O_2_)^27,28^. The activity of LAO has been identified in several different cell types, such as mammary gland, liver, and kidney cells, as well as polymorphonuclear leukocytes^29,30^. Mammals have two related molecules encoded by the genes *LAO1* and *interleukin 4-induced gene 1* (*IL4i1*), which have different properties in the above-mentioned cells.

It should be noted that mice have both *LAO1* and *IL4i1*, while *LAO1* has been lost in humans, leaving *IL4i1* as the apparent sole gene with LAO activity^31^. In our preliminary studies, we found that the mRNA for *LAO1* is expressed in the mouse brain. Furthermore, we observed that compared to wild-type (WT) mice, *LAO1* knockout (KO) mice exhibited memory impairment, which suggested that further analysis of brain function in *LAO1* KO mice might improve our understanding of the mechanisms involved in abnormal amino acid metabolism-related brain dysfunction. Therefore, in this study, we first conducted several behavioural tests to confirm the presence of brain dysfunction in *LAO1* KO mice. Second, we measured the amount of free amino acids and their metabolites in the plasma and hippocampus using metabolomic analyses to identify the key molecule responsible for inducing the brain dysfunction. Finally, we measured the levels of several neurotransmitters, including acetylcholine, dopamine, and serotonin, in the hippocampus, to determine the effects of donepezil on brain function in *LAO1* KO mice.

## Results

### Impairment of fear learning and memory in *LAO1* KO mice

In the passive avoidance (PA) test, mice with normal fear learning and memory avoid entering the dark compartment after being exposed to a foot shock on training. There were no differences in the time taken to enter the dark compartment between WT and *LAO1* KO mice on the training. To ensure the animals’ responses to painful stimuli did not differ, we first performed the hot plate test; no differences in the sensitivity to painful stimuli were identified between WT and *LAO1* KO mice (Supplementary Fig. S1). As for the PA test, *LAO1* KO mice were less avoidance entering the dark compartment than did WT mice at 3 h after the foot shock (Fig. 1b). This finding suggests that the depletion of *LAO1* decreased the mice’s learning ability and/or short-term memory against fear conditioning. To evaluate long-term memory, the mice’s avoidance at longer intervals (1 and 3 days) post-foot shock was evaluated using the mice which had avoided entering the dark compartment at 3 h (Fig. 1c). The *LAO1* KO mice exhibited a tendency to re-enter the dark compartment when compared to WT mice and there was significant difference at 3 days after the foot shock, suggesting that long-term memory might also be impaired in *LAO1* KO mice. No significant differences were identified between WT and *LAO1* KO mice in terms of their emotional- (open field test) and anxiety-like behaviours (elevated plus maze test) (Fig. 1d–f).

### Concentration of free amino acids in the plasma of *LAO1* KO mice

**Figure 2:**
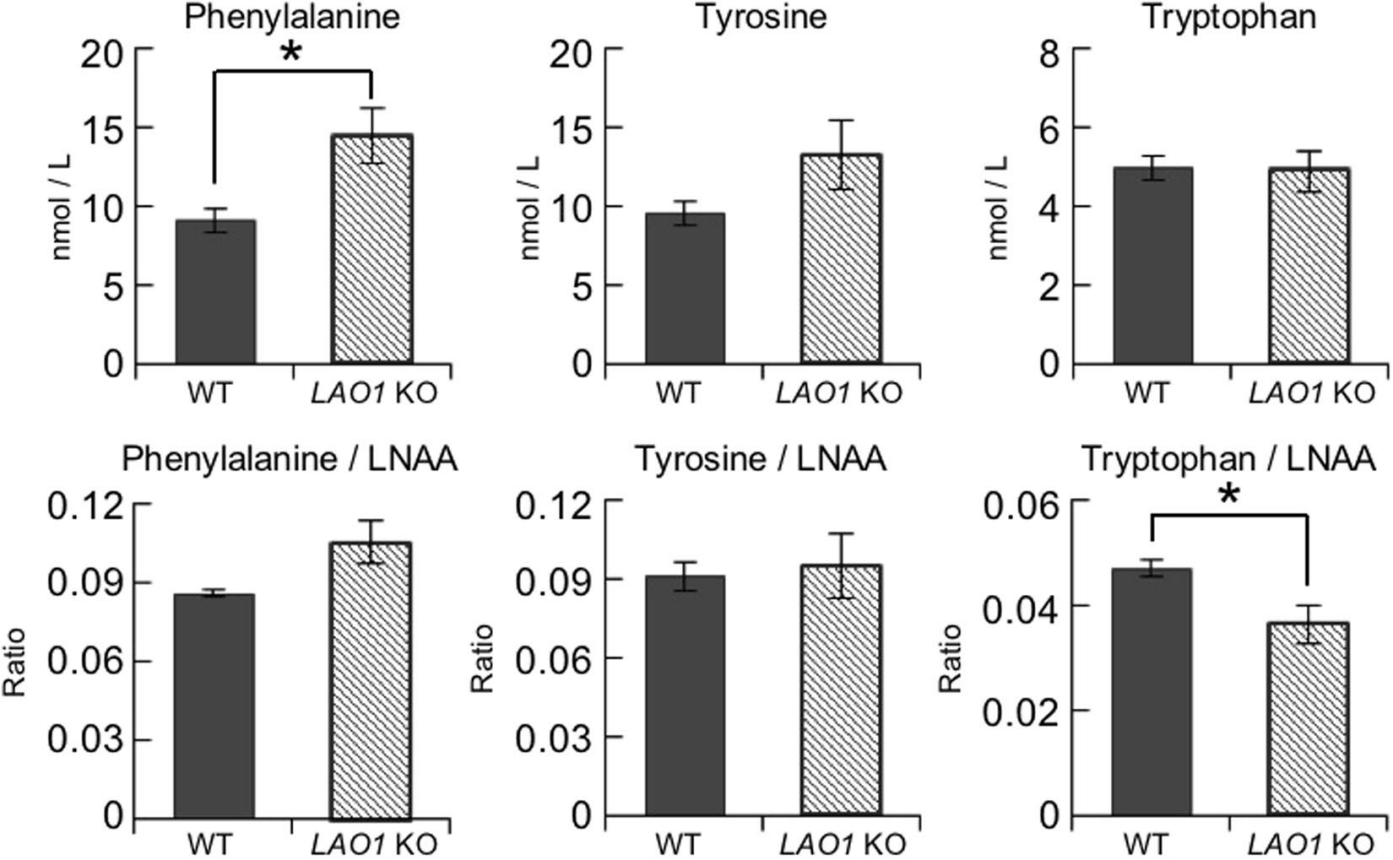
Concentrations of free amino acids in the plasma of *LAO1* KO mice. The concentrations of 19 L-amino acids in the plasma of male WT (n = 6) and *LAO1* KO (n = 6) mice were measured using the EZ:faast GC-MS Kit. The histogram displays Phe, Tyr, and Trp concentrations (top), and Phe/LNAA, Tyr/LNAA, and Trp/LNAA ratios (bottom).

We performed an amino acid analysis using gas chromatography-mass spectrometry (GC-MS) to investigate the free plasma amino acid concentration profiles of WT and *LAO1 KO* mice (Fig. 2 and Supplementary Table S1). The plasma concentration of Phe was significantly higher in *LAO1* KO mice than it was in WT mice. Since large neutral amino acids (LNAAs), including Phe, compete for transport across the blood-brain barrier, the concentration of each amino acid relative to the total concentration of LNAAs is an important parameter that is often used to evaluate the uptake of each amino acid into the brain^32^. Here, we found that the relative proportions of Phe and Tyr, two sources of catecholamines, to the amount of total LNAAs were not different between *LAO1* KO and WT mice (Fig. 2). However, the ratio of Trp, a source of serotonin, to LNAAs was significantly lower in the plasma of *LAO1* KO mice than it was in the plasma of WT mice, implying that *LAO1* KO mice may have low levels of serotonin in the brain.

### Contribution of hippocampal neurotransmitters to contextual fear memory in *LAO1* KO mice

**Figure 3:**
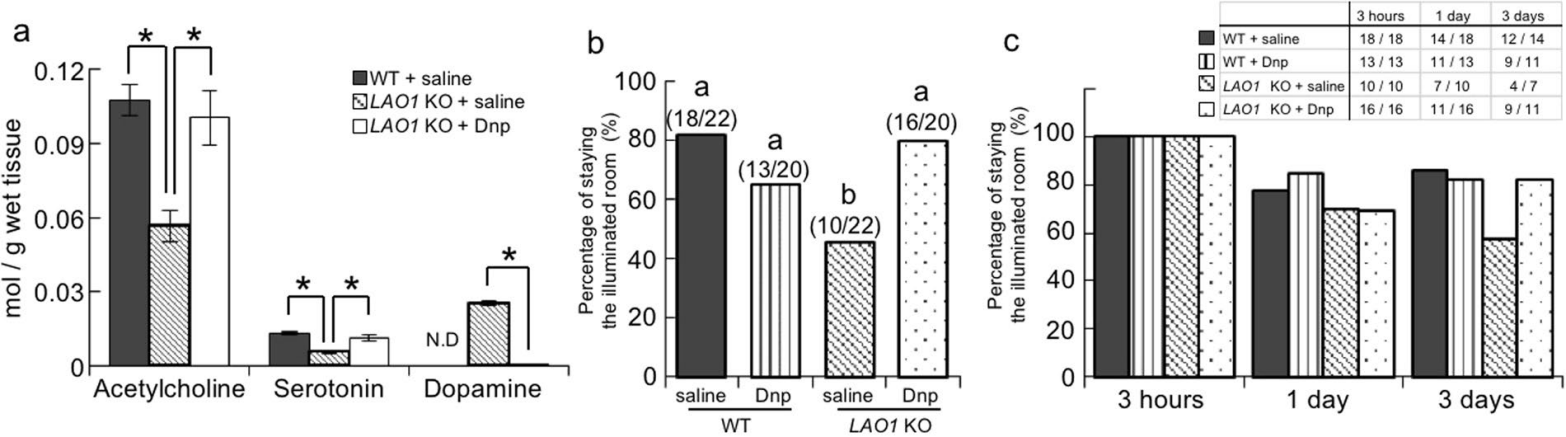
Contributions of hippocampal neurotransmitters to contextual fear memory in *LAO1* KO mice. (**a**) The concentrations of acetylcholine, serotonin, and dopamine in the hippocampus of male WT mice treated with saline, *LAO1* KO mice treated with saline, and *LAO1* KO mice treated with donepezil were measured using LC-MS/MS. (**b**,**c**) Passive avoidance test. Adult male WT mice treated with saline (n = 22), WT mice treated with donepezil (n = 20), *LAO1* KO mice treated with saline (n = 22), and LAO1 KO mice treated with donepezil (n = 20) received a foot shock in the dark compartment on the first day. The results represent the percentage of time spent avoiding the dark compartment by the mice at 3 h, 1 and 3 days after the foot shock.

To further examine the neural underpinnings of the memory impairments, we measured the levels of the typical neurotransmitters acetylcholine, serotonin, and dopamine. We observed lower acetylcholine and serotonin concentrations and higher dopamine levels in the hippocampus of *LAO1* KO mice vs. WT mice (Fig. 3a). To investigate the involvement of the reduced acetylcholine levels in the contextual fear memory of *LAO1* KO mice, one group of *LAO1* KO mice was pre-treated with donepezil before undergoing the PA test. Treating the *LAO1* KO mice with donepezil restored the hippocampal concentrations of acetylcholine, serotonin, and dopamine to the levels found in WT mice (Fig. 3a). In the PA test, *LAO1* KO mice treated with saline were less avoidance entering the dark compartment than did WT mice with saline at 3 h after the foot shock (Fig. 3b). Treating the *LAO1* KO mice with donepezil elevated their score on the PA test at 3 h to levels that were similar to those of WT mice with saline. At 3 days after the foot shock, the *LAO1* KO mice treated with donepezil also avoided entering the dark compartment when compared to *LAO1* KO mice with saline, although the differences were not significant (Fig. 3c).

### Changes in the hippocampal levels of amino acid metabolites in *LAO*1 KO mice

**Figure 4:**
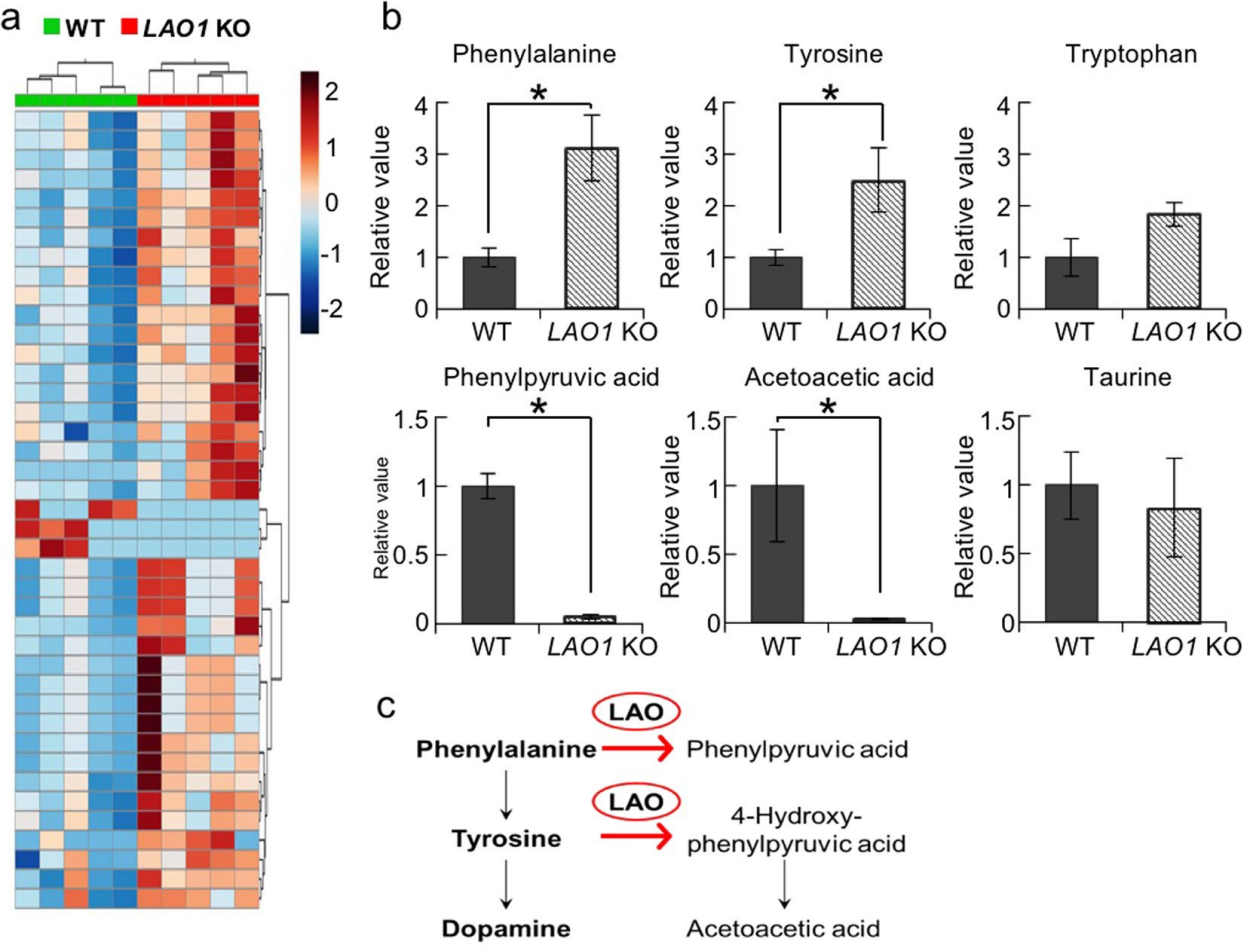
Changes in amino acid metabolites in the hippocampus of *LAO1* KO mice. (**a**) Heat map of the relative significant changes in the hippocampus of male WT and *LAO1* KO mice (n = 5 per group). The heat map ranges from −2 to +2 on a log2 scale. (**b**) Histograms of typical metabolite changes between male WT and *LAO1* KO mice (n = 5 per group). (**c**) Schematic diagram showing Phe metabolism. LAO is the metabolic enzyme that converts Phe to phenylpyruvic acid and Tyr to acetoacetic acid via 4-hydroxy-phenylpyruvic acid.

An untargeted metabolomic analysis of the hippocampus revealed that the levels of 41 metabolites were significantly different between WT and *LAO*1 KO mice (Fig. 4a and Supplementary Table S2). Because LAO is an amino acid metabolism enzyme, predictably, the levels of some amino acids were significantly higher in *LAO1* KO mice than they were in WT mice. In particular, the Phe and Tyr levels, which are major substrates for LAO, were significantly higher in the hippocampus of *LAO1* KO mice (Phe: *p* = 0.012, Tyr: *p* = 0.036) (Fig. 4b). However, the levels of the metabolites phenylpyruvic acid and acetoacetic acid were significantly lower in *LAO1* KO mice than they were in WT mice (phenylpyruvic acid: *p* = 0.013, acetoacetic acid: *p* = 0.044) (Fig. 4b). This finding suggests the presence of a block in Phe/Tyr metabolism in the hippocampus *LAO1* KO mice (Fig. 4c). No significant difference in the concentration of Trp was identified between *LAO1* KO and WT mice (Fig. 4b).

### Decreased hippocampal *PK* mRNA levels in *LAO1* KO mice

**Figure 5:**
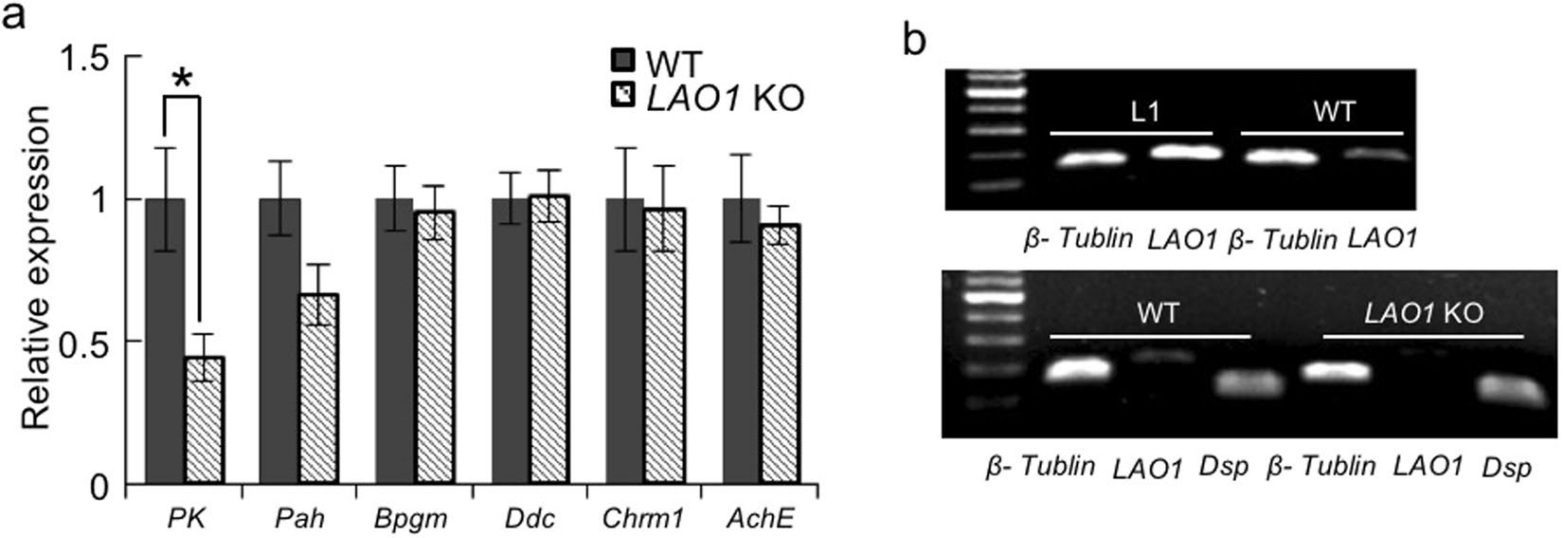
Reduction in hippocampal *PK* mRNA levels in *LAO1* KO mice. (**a**) Comparison of *PK*, *Pah*, *Bpgm*, *Ddc*, *Chrm1*, and *AchE* expression in the hippocampus using real-time PCR. WT: hippocampus in male WT mice (n = 7), *LAO1* KO: hippocampus in male *LAO1* KO mice (n = 7). (**b**) *LAO1* mRNA expression in the hippocampus. L1: mammary gland at lactation day 1 in female WT mice (used as a positive control), WT: hippocampus in male WT mice, LAO1 KO: hippocampus in male LAO1 KO mice. *β-tubulin* was used as a housekeeping gene and *Dsp* was used as a neuron-specific gene.

Phe accumulation reportedly leads to the inhibition of pyruvate kinase (PK) expression in the brains of patients with PKU^33^. Consistent with this observation, our gene expression analysis indicated that compared to WT mice, *LAO1* KO mice exhibited significantly lower *PK* mRNA expression in the hippocampus (Fig. 5a). No significant differences in the gene expression levels of *Pah*, *bisphosphoglycerate mutase* (a gluconeogenesis enzyme: *Bpgm*), *aromatic L-amino acid decarboxylase* (*Ddc*), *muscarinic M1 acetylcholine receptor* (*Chrm1*), and *AchE* were found between *LAO1* KO and WT mice (Fig. 5a). We also confirmed the presence of *LAO1* mRNA expression in the hippocampus of WT mice by using a lactating mammary gland (L1) obtained from female WT mice as a positive control (Fig. 5b).

## Discussion

To the best of our knowledge, the present study is the first to show that local LAO in the hippocampus is important for brain function, as it regulates Phe metabolism in mice. Behavioural tests indicated that *LAO1* KO mice have impairments in fear learning and memory compared to WT mice. Moreover, these impairments appear to be specific to hippocampus-dependent memory, because the locomotion- and anxiety-like behaviours of *LAO1* KO mice were not different from those of WT mice. Our findings suggest that reductions in the levels of hippocampal acetylcholine are closely related to the memory impairments we identified in *LAO1* KO mice. This is supported by the observation that *LAO1* KO mice pre-treated with donepezil had normal memory function and a hippocampal acetylcholine level that was similar to the level we observed in WT mice. Moreover, our metabolomic analysis revealed high concentrations of Phe in the plasma and hippocampus of *LAO1* KO mice. It should be noted that the accumulation of Phe in the hippocampus was not due to the uptake of plasma Phe into the brain, as the concentration of Phe relative to that of total LNAAs was not significantly different between WT and *LAO1* KO mice. We also confirmed the changes in *LAO1* gene expression in the hippocampus using real-time polymerase chain reaction (PCR) analyses. Collectively, our data indicated that the local metabolism of Phe by LAO might affect brain function by regulating acetylcholine synthesis.

It is known that increased concentrations of Phe in the plasma and brain lead to brain abnormalities and functional impairment in patients with PKU^34^. Children with PKU, if untreated, have severe and irreversible intellectual disabilities in working memory and attentional control^35^. In addition, these disabilities are not apparent in younger children with PKU and are observed only in older children, suggesting the presence of a developmental deficit rather than a developmental delay in the working memory of children with PKU^36^. The toxic effects of accumulated Phe in the brain can be minimised by following a low-Phe diet^37^. However, this diet is very restricted (Phe is present in most foods containing protein), thus it is difficult to control the levels of Phe consumed in food after weaning. Furthermore, one study that evaluated the working memory, sustained attention, and inhibition of children with PKU found executive dysfunction despite the presence of normal intelligence quotients in these children, even during continuous dietary treatment^38^. Currently, the precise mechanisms responsible for the neurological effects of Phe accumulation are unclear. Based on our results, we propose that the accumulation of Phe is neurotoxic and may inhibit the glycolytic pathway and reduce acetylcholine synthesis, which results in impairments in learning and memory functions.

Acetylcholine is synthesised in nerve cells by choline acetyltransferase from choline and acetyl coenzyme A. Acetyl coenzyme A is in turn generated from pyruvate. We found that the mRNA levels for *PK*, which leads to the production of adenosine triphosphate and pyruvate in the final step of glycolysis, were reduced in the hippocampus of *LAO1* KO mice when compared to that of WT mice. This finding implies that the low levels of *PK* mRNA might also lead to reductions in the levels of molecules produced in glycolysis, namely those required for acetylcholine synthesis, ultimately inhibiting acetylcholine synthesis in nerve cells. In addition, because PK plays a crucial role in glycolysis and brain function, the lower expression of *PK* mRNA we observed may be related to reduced glucose metabolism, as well as to the inhibition of acetylcholine synthesis in the hippocampus of *LAO1* KO mice. Given these observations, we hypothesise that administering a drug that controls the above pathway, e.g. donepezil, may be useful for reducing the risk for and/or severity of mental disorders in patients with PKU. Further experiments using *LAO1* KO and *Pah* KO mice are required to prove our hypothesis.

**Figure 6:**
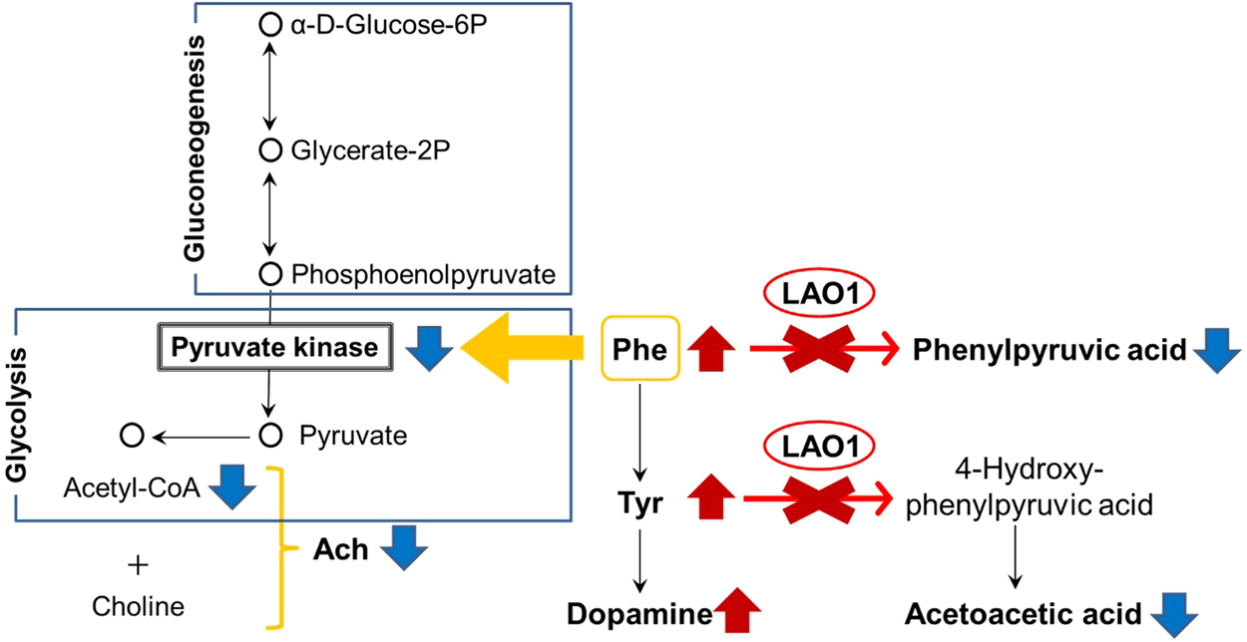
Schematic diagram showing metabolomic changes observed in *LAO1* KO mice. In the hippocampus of *LAO1* KO mice, Phe and Tyr accumulation occurs owing to dysfunction in Phe metabolism. High levels of Tyr lead to elevated dopamine and the accumulation of Phe inhibits *PK* mRNA expression. Reduced levels of *PK* affect glycolysis and decrease acetyl coenzyme A synthesis. Finally, low levels of acetylcholine are observed in the hippocampus of *LAO1* KO mice.

In the present study, we found that like acetylcholine, the concentration of serotonin was reduced in the hippocampus of *LAO1* KO mice compared to that of WT mice. In contrast, the level of dopamine was elevated in *LAO1* KO mice when compared to WT mice. Serotonin and dopamine are multifunctional neurotransmitters that are present in the cortical and limbic regions, two areas that are involved in cognition and emotional regulation^39,40^. Therefore, the production of serotonin from Trp and that of dopamine from Tyr are important metabolic pathways in the brain^40^. Here, our real-time PCR analyses did not reveal a difference in hippocampal *Ddc* mRNA expression between WT and *LAO1* KO mice. *Ddc* encodes a protein that catalyses the decarboxylation of L-3,4-dihydroxyphenylalanine to dopamine and that of L-5-hydroxytryptophan to serotonin, among other reactions. Because the synthesis of these monoamines in the hippocampus partly depends on the concentrations of the respective precursor amino acids, the elevated dopamine levels we observed in *LAO1* KO mice may be related to the accumulation of Phe and Tyr following the depletion of *LAO1* expression in the hippocampus (Fig. 6). The most important factor controlling the brain uptake of Trp, an important factor for serotonin synthesis, from the circulation is its competition with other LNAAs, including valine, leucine, isoleucine, methionine, histidine, Phe, Tyr, and Trp, for transport to the brain across L-amino acid transporter-1 (SLC7A5) at the blood-brain barrier^41^. In our study, the relative Trp to LNAA ratio was reduced in the plasma of *LAO1* KO mice compared to the ratio in WT mice. This finding implies that the lower level of serotonin in the hippocampus may be due to a shortage of Trp in the neurons of the raphe nuclei, which are the principal sources of serotonin in the brain^42^. Interestingly, treating *LAO1* KO mice with donepezil altered the hippocampal concentrations of not only acetylcholine but also serotonin and dopamine to levels that were similar to those found in WT mice. Unfortunately, the mechanisms responsible for the above effects of donepezil are unclear. As such, further experiments are required to investigate the hierarchy of these neurotransmitters and the contribution of each to brain processes such as learning and memory in *LAO1* KO mice.

The activity of LAO, which comprises the oxidation of L-amino acids, has been widely observed in several tissues, such as the brain, mammary glands, kidneys, liver, and testes, as well as in immune cells^28–30^. To date, however, the biological significance of this activity, especially with regard to brain function, has not been elucidated. *IL4i1*, a homolog of *LAO1*, is expressed by immune cells, including macrophages, T cells, and B cells, upon stimulation by IL4, and has been shown to exert immunomodulatory functions in various tumours and in response to bacterial infections^43–46^. Here, we found that male *LAO1* KO mice have deficits in fear memory, as assessed using the PA test. Intriguingly, the Mouse Genome Informatics database indicates that male *IL4i1* KO mice have enhanced learning and memory during contextual fear conditioning when compared to male WT mice. Moreover, a recent study demonstrated that *IL4i1* expression is induced in central nervous system lesions and is involved in myelin regeneration (remyelination) in mice^47^. These observations suggest that LAO activity is involved in not only learning and memory but also repair processes in the brain. Further studies using conditional and/or double (*LAO1* and *IL4i1*)-KO mice are needed to determine the detailed mechanisms.

In conclusion, the findings from our study suggest that *LAO1* is important for modulating hippocampal neurotransmitter levels, which contribute to the learning and memory of contextual fear conditioning in mice. The hippocampal expression of *LAO1* affects acetylcholine and dopamine synthesis by locally regulating Phe metabolism. Additionally, *LAO1* controls and decreases the ratio of Trp to LNAA in the plasma of *LAO1* KO mice, subsequently affecting serotonin synthesis in the brain. However, as mentioned above, mice have both *LAO1* and *IL4i1*, while *LAO1* has been lost in humans, leaving *IL4i1* as the apparent sole gene with LAO activity^31^. To understand the exact functions of LAO activity in humans, additional analyses using *LAO1* KO mice and *IL4i1* KO mice will be necessary in the future.

## Methods

### Animals

Male *LAO1* KO mice backcrossed to C57BL/6 J mice were housed 4–5 per cage containing wood-shaving bedding at 23 ± 2 °C under a 14-h lighting schedule (lights on 05:00 to 19:00) with free access to food (MR-Breeder, Nosan Corporation, Yokohama, Kanagawa, Japan) and tap water. Ten-week-old male mice were used for the behavioural test battery, which consisted of open field, elevated plus maze (male WT mice: n = 22, male *LAO1* KO mice: n = 22), and PA tests (male WT mice: n = 30, male *LAO1* KO mice: n = 30). Before starting behaviour test, animals were handled for 5 min daily for 3days. The intervals between these tests were at least 1 day. The apparatuses were cleaned with 70% ethanol to ensure that no cue smell remained from the previous trial. After the test battery was completed, mice were sacrificed by cervical dislocation. Following this, plasma and hippocampal tissue were collected and stored at −80 °C until use. For the experiments involving donepezil treatment, the mice were separated into four groups and orally treated with donepezil (1 mg/kg) or saline (male WT mice + saline: n = 28, male WT mice + donepezil: n = 26, male *LAO1* KO mice + saline: n = 28, and male *LAO1* KO mice + donepezil: n = 26). After 1 h, the mice were used for the behavioural tests (male WT mice + saline: n = 22, male WT mice + donepezil: n = 20, male *LAO1* KO mice + saline: n = 22, and male *LAO1* KO mice + donepezil: n = 20) or hippocampus collection (n = 6 per group). All experiments were performed during the light period and were approved by the Animal Care and Use Ethical Committee of Tokyo University of Agriculture and Technology (approval number: 24–80).

### Behavioural tests

#### Open field test

The open field test was used to evaluate locomotor activity and emotional responses^48^. The apparatus was a transparent square cage (40 × 40 × 30 cm). The centre of the floor was illuminated at 20 lux. Each mouse was placed in the open field apparatus and recorded for 10 min. The average velocity (cm/s) and time spent in the centre area (20 × 20 cm) were measured by EthoVision XT 10 (Noldus Information Technology, Inc., Leesburg, VA, USA).

#### Elevated plus maze test

The elevated plus maze test is used to investigate anxiety-like behaviour in rodents^49^. The elevated plus maze consisted of two open arms (25 × 5 cm with 3 cm-high ledges) and two closed arms (25 × 5 cm with 30 cm-high transparent walls) of the same size. The arms and central square were made of white plastic plates and were elevated to a height of 55 cm above the floor. Arms of the same type were arranged opposite to each other. The centre of the maze was illuminated at 20 lux. Each mouse was placed in the central square of the maze (5 × 5 cm) facing one of the closed arms and was recorded for 10 min. The number of total entries into the open and closed arms and time spent in the open and closed arms were calculated by EthoVision XT10.

#### Passive avoidance test

The contextual learning PA test was conducted as previously described^50^. The step-through PA apparatus consisted of a box divided into two compartments. One compartment (15 × 10 × 10 cm) was illuminated with a 200-lux lamp placed at the top of the chamber and the other was dark. The compartment was separated by a guillotine door (10 × 12 cm). On the first day, each mouse was placed in the illuminated safe compartment. The mice tended to escape to the dark compartment where they received a constant-voltage (0.3 mA) foot shock for 3 s. After the trial, the mice were returned to their home cages. The PA test was repeated 3 h, and 1 and 3 days later, and the percentage of mice number spent in the light compartment during a 5-min period was calculated. Once the mice entered the dark compartment, they were omitted at further time points.

#### Hot plate test

The hot plate test was performed as previously described to check the animals’ responses to thermal stimuli^51^. Mice (male WT mice: n = 8, male *LAO1* KO mice: n = 8) were placed on a hot plate preheated to 55 °C until the mice manifested a nociceptive behaviour (lifting or licking its hind-paw).

### Metabolomic analysis

The levels of neurotransmitters in the hippocampus were measured using liquid chromatography-tandem mass spectrometry (LC-MS/MS; ACQUITY TQD UPLC-MS/MS, Waters, Milford, MA, USA). In brief, fresh hippocampal samples were homogenised in 0.2 M perchloric acid (500 μL per 100 mg wet tissue) and kept in a dark cool place. After 30 min, the homogenates were centrifuged at 20,000 × *g* at 0 °C for 15 min. The supernatants, which were filtrated using a 0.2-μm filter, were used for the measurements. The analytes were separated on a Scherzo SS-C18 column (150 × 3 mm, 3 μm; Imtakt Corp., Kyoto, Kyoto, Japan) with the column temperature set at 40 °C. The mobile phase comprised solvent A (methanol/water/formic acid = 10/90/0.5) and solvent B (methanol/100 mM ammonium formate = 30/70). The mobile phase was eluted at 0.6 mL/min according to the gradient as follows: 100% solvent A maintained for 3.5 min, decreased to 0% at 6 min, and held for 4 min. The ESI source was operated in the positive ionisation mode, and its main working parameters were set as follows: gas temperature, 500 °C; ionisation heater temperature, 150 °C; desolvation gas flow, 900 L/h; cone gas flow rate, 250 L/h; and capillary voltage, 3000 V. The internal standard was a stable tyrosine isotope (13C15N). The multiple reaction monitoring transitions and individual parameters that were applied for the analytes are summarised in Supplementary Table S3.

Plasma samples were deproteinised using an Amicon Centrifree system (10 kDa; Merck Millipore, Darmstadt, Germany), and their free amino acid concentrations were measured using the EZ:faast GC-MS Kit (Phenomenex Inc., Torrance, CA, USA) for the amino acid analysis. As a standard solution, Amino Acids Mixture Standard Solution (Type AN-2 and B) and solutions of L-tryptophan, L-asparagine, and L-glutamine (Wako Pure Chemical Industries Ltd., Chuo, Osaka, Japan) were used. Norvaline solution (0.2 mM) in the kit was used as an internal standard. The procedure requires a concentration step on a proprietary sorbent medium, elution from and removal of the sorbent medium, and sample clean-up, as well as derivatisation with a proprietary chloroformate derivative. The derivatised samples were analysed using GC-MS (GCMS QP2010-Ultra, Shimadzu Corp., Kyoto, Kyoto, Japan).

An untargeted metabolomics analysis was performed using GC-MS, as described previously, with some modifications^52^. In brief, frozen hippocampal samples were suspended in 250 μL methanol-chloroform-water (2.5:1:1) and 5 μL of 1 mg/mL 2-isopropylmalic acid as an internal standard, and homogenised using a Polytron homogeniser (Micro-tec Co., Ltd., Urayasu, Chiba, Japan). Samples were subsequently mixed in a shaker at 1200 rpm at 37 °C for 30 min and centrifuged at 16,000 × *g* at 4 °C for 5 min. Next, 160 μL of the supernatant was mixed with 200 μL of distilled water and vortexed. This was followed by centrifugation at 16,000 × *g* at 4 °C for 5 min. Afterwards, 250 μL of the supernatant was dried under a vacuum using a centrifugal evaporator (RD-400, Yamato Scientific Co., Ltd., Koto, Tokyo, Japan). Dried samples were pre-treated, derivatised, and analysed using GC-MS (GCMS QP2010-Ultra, Shimadzu) within 24 h of derivatisation, as described^52^. The Shimadzu Smart Metabolites Database was used to identify metabolites.

### Quantitative real-time PCR

Total RNA was extracted from the hippocampus using ISOGEN II Reagent (Nippon Gene Co., Ltd., Chiyoda, Tokyo, Japan), according to the manufacturer’s protocol. cDNA was synthesised using the Prime Script 1st strand Complementary DNA Synthesis Kit (Takara Bio Inc., Kusatsu, Shiga, Japan). The oligonucleotide primers for the quantitative real-time PCR analysis were designed using the Primer 3 program (Supplementary Table S4). The PCR reactions were performed at a volume of 10 μL using Ex Taq Hot Start Version containing SYBR-Green I (Takara Bio) and the Chrome4 real-time PCR System (Bio-Rad, Hercules, CA, USA) using the following conditions: 95 °C for 30 s followed by 40 cycles of 95 °C for 5 s, 60 °C for 30 s, and dissociation. The relative expression level of each target mRNA relative to tubulin mRNA was determined using the 2^−ΔΔ^CT method.

### Statistical analysis

All data were analysed with GraphPad Prism (National University of Ireland Galway, University Road, Galway, Ireland) and SPSS statistics (IBM Corporation, Armonk, NY, USA). Values are expressed as the mean ± standard error of the mean. Unpaired *t*-tests were used to detect differences between WT and *LAO1* KO mice in the open field test, elevated plus maze test, metabolomic analysis, and quantitative real-time PCR analysis. Fisher’s exact test was used to detect group differences in the PA test. In the experiment of donepezil treatment, one-way ANOVA analysis of variance and Tukey post hoc testing was used to assess statistical significance of neurotransmitter measurement. Also, Chi-squared Test and the residual analysis were performed to identify significant differences in PA test after donepezil treatment. Differences were considered significant at *p* < 0.05.

### Data availability

The datasets generated and/or analysed during the current study are available from the corresponding author on reasonable request.

## Electronic supplementary material

Supplementary Information
