# Supplementary material for: Hippocampal metabolism of amino acids by L-amino acid oxidase is involved in fear learning and memory
Source: Sci Rep. 2018 Jul 23;8:11073. doi: 10.1038/s41598-018-28885-x (PMC6056520; doi:10.1038/s41598-018-28885-x)
Supplement: Supplementary file 1 — Supplementary Information [file 41598_2018_28885_MOESM1_ESM.pdf]

Supplementary information

Hippocampal metabolism of amino acids by L-amino acid oxidase is involved in fear learning and memory

Kento Usuda, Takahiro Kawase, Yuko Shigeno, Susumu Fukuzawa, Kazuki Fujii, Haolin Zhang, Takamitsu Tsukahara, Shozo Tomonaga, Gen Watanabe, Wanzhu Jin & Kentaro Nagaoka

Conntent

Figure S1. Hot plate test.

Figure S2. Original gel image in Fig. 5

Table S1. The result of measurement of 19 L-amino acids in plasma.

Table S2. The list of metabolites that showed significant difference between WT and LAO KO hippocampus.

Table S3. Multiple reaction monitoring transitions and individual parameters for the neurotransmitters analysis by LC-MS/MS.

Table S4. The list of primers.

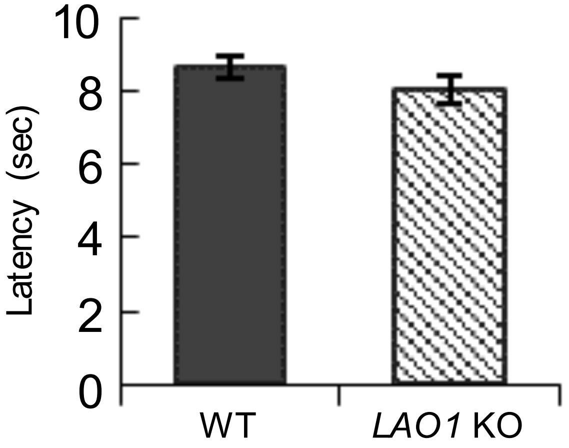

Figure S1. Hot plate test. Mice (male WT mice: n=8, male LAO1 KO mice: n=8) were placed on a hot plate preheated to 55° C until the mice manifested a nociceptive behaviour

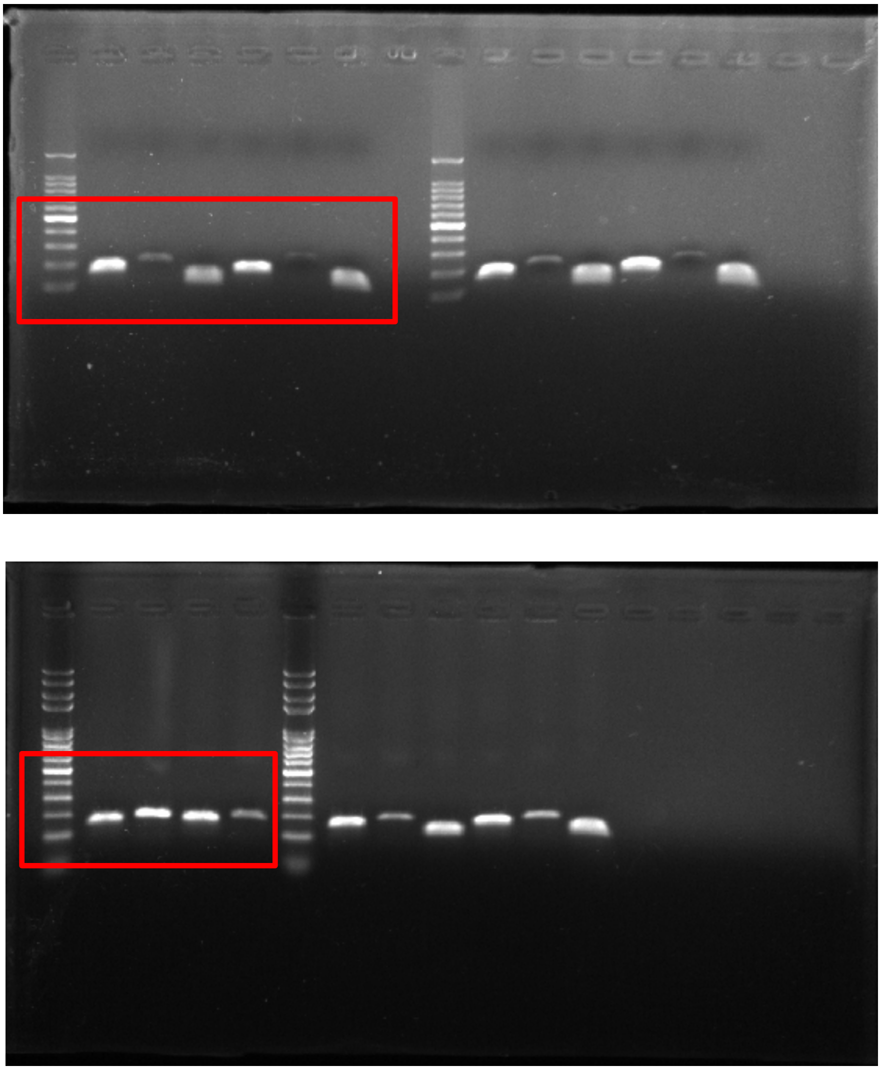

Figure S2. Original gel image in Fig. 5.

	WT	LAO KO	p value
Alanine	39.23 ± 3.03	50.21 ± 2.34	p < 0.05
Glycine	41.05 ± 2.41	48.93 ± 2.84	N.S
Valine	31.04 ± 2.28	39.58 ± 2.75	p < 0.05
Leucine	21.79 ± 1.91	28.27 ± 2.19	N.S
Isoleucine	12.17 ± 1.02	14.68 ± 1.10	N.S
Threonine	20.59 ± 2.35	26.39 ± 2.27	N.S
Serine	14.15 ± 1.27	19.03 ± 2.00	N.S
Proline	16.56 ± 1.21	22.33 ± 1.78	p < 0.05
Asparagine	5.76 ± 0.80	10.10 ± 1.48	p < 0.05
Aspartic acid	2.89 ± 0.28	4.01 ± 0.44	N.S
Methionine	8.76 ± 0.60	11.08 ± 0.86	N.S
Glutamic acid	10.04 ± 0.80	11.88 ± 0.88	N.S
Phenylalanine	9.15 ± 0.74	14.45 ± 1.75	p < 0.05
Glutamine	39.53 ± 3.34	57.57 ± 8.00	N.S
Ornithine	11.44 ± 0.90	16.66 ± 1.88	p < 0.05
Lysine	28.19 ± 2.84	34.97 ± 2.82	N.S
Histidine	8.36 ± 0.12	9.55 ± 0.65	N.S
Tyrosine	9.61 ± 0.78	13.23 ± 2.23	N.S
Tryptophan	4.97 ± 0.31	4.91 ± 0.52	N.S
LNAA	105.84 ± 7.13	135.75 ± 8.75	p < 0.05
Phe/LNAA	0.086 ± 0.016	0.106 ± 0.008	N.S
Tyr/LNAA	0.091 ± 0.006	0.095 ± 0.012	N.S
Trp/LNAA	0.047 ± 0.002	0.036 ± 0.004	p < 0.05

Table S1. The result of measurement of 19 L-amino acids in plasma.
Data are presented as means (nmol / L) ± S.E.M. $p < 0.05$, unpaired t-test.
N.S: no significant difference.

	WT	LAO KO
Mannitol	1 ± 0.18	1.73 ± 0.19
Arabitol	1 ± 0.16	1.62 ± 0.20
Malic acid	1 ± 0.13	1.99 ± 0.26
Isocitric acid&Citric acid	1 ± 0.15	2.01 ± 0.29
5-Hydroxyindoleacetic acid	1 ± 0.11	2.04 ± 0.14
Erythritol	1 ± 0.15	1.95 ± 0.13
Uracil	1 ± 0.18	2.12 ± 0.21
Niacinamide	1 ± 0.16	1.74 ± 0.11
Lyxose	1 ± 0.17	1.78 ± 0.17
Fumaric acid	1 ± 0.17	1.71 ± 0.23
(+/-)-erythro-Isoleucine	1 ± 0.16	2.03 ± 0.20
N-Acetylglutamic acid	1 ± 0.13	1.92 ± 0.22
Beta-Alanine	1 ± 0.23	2.01 ± 0.24
O-Phosphoethanolamine	1 ± 0.17	2.14 ± 0.31
Adenine	1 ± 0.14	1.94 ± 0.22
Glycine	1 ± 0.19	2.04 ± 0.28
Indoxyl sulfate	1 ± 0.32	2.30 ± 0.28
Galactitol	1 ± 0.15	2.13 ± 0.44
Dopamine	1 ± 0.18	2.13 ± 0.28
Hypotaurine	1 ± 0.16	2.00 ± 0.26
Acetoacetic acid	1 ± 0.41	0.02 ± 0.005
Alpha-ketoisovaleric acid	1 ± 0.41	0.004 ± 0.0007
Phenylpyruvic acid	1 ± 0.09	0.05 ± 0.013
Sarcosine	1 ± 0.16	2.00 ± 0.26
2-Aminoisobutyric acid	1 ± 0.16	2.00 ± 0.26
Phenylalanine	1 ± 0.17	3.12 ± 0.64
Cadaverine	1 ± 0.31	3.91 ± 1.18
3-Hydroxybutyric acid	1 ± 0.17	2.62 ± 0.59
5-Sulfosalicylic acid	1 ± 0.16	2.35 ± 0.53
threo-3-Hydroxy-aspartic acid	1 ± 0.16	2.35 ± 0.53
N-Formyl-L-methionine	1 ± 0.16	2.35 ± 0.53
2-Methyl-1-propylamine	1 ± 0.16	2.55 ± 0.48
Deoxyguanosine	1 ± 0.16	2.55 ± 0.48
Pelargonic acid	1 ± 0.27	2.49 ± 0.42
Homoserine	1 ± 0.17	1.76 ± 0.21
Serine	1 ± 0.17	1.62 ± 0.20
Methionine sulfone	1 ± 0.99	6.57 ± 1.76
N-Acetyl-b-D-galactosamine	1 ± 0.36	2.18 ± 0.20
Glutamic acid	1 ± 0.29	2.02 ± 0.14
Allantoin	1 ± 0.37	2.02 ± 0.19
Tyrosine	1 ± 0.16	2.49 ± 0.55

Table S2. The list of metabolites that showed significant difference between WT and LAO KO hippocampus.

Analytes	m / z	Cone voltage (V)	Collision Energy (V)
Tyrosine $^{13}C_9$, $^{15}N_1$	192.3 > 145.2	25	18
Acetylcholine	146.0 > 87.2	25	18
Serotonin	177.1 > 160.2	18	18
Dopamine	154.0 > 137.2	24	18

Table S3. Multiple reaction monitoring transitions and individual parameters for the neurotransmitters analysis by LC-MS/MS.

Primer	Sequence 5' to 3'
PK Forward	ggtgtttgcatctttcatcc
PK Reverse	ccttctctgcaggaatctca
Pah Forward	ccacccaggctttaaagatcc
Pah Reverse	cagagtcctgaacaccgttcc
Bpgm Forward	taatttatcgtggcccagacg
Bpgm Reverse	acgggaggagcagaaaagttc
Ddc Forward	caggcttacatccgaaagcac
Ddc Reverse	agcagaccaacccaagaatga
Chrm1 Forward	accagcatcaggaccggaaa
Chrm1 Reverse	tttgccgcatccctctgaca
AchE Forward	cactcctcttcctcctcctc
AchE Reverse	gtggtagcatccaacactcc
LAO1 Forward	tggccaagaagagtggaatc
LAO1 Reverse	agctcccactaccaccacac
β-Tublin Forward	gctgaccagtgcacgc
β-Tublin Reverse	aaacctggggggctgggt
Dsp Forward	gctgaagaacactctagccca
Dsp Reverse	actgctgtttcctctgagaca

Table S4. The list of primers.

PK: *Pyruvate kinase*, *Pah*: *Phenylalanine hydroxylase*, *Bpgm*: *2,3-bisphosphoglycrate mutase*, *Ddc*: *Aromatic L-amino acid decarboxylase*, *Chrm1*: *muscarinic M1 Ach receptor*, *AchE*: *Acetylcholine esterase*, *Dsp*:*desmoplakin*
